# Sodium bicarbonate prophylactic therapy in the prevention of contrast-induced nephropathy in patients admitted to the intensive care unit of a teaching hospital: a retrospective cohort study

**DOI:** 10.1186/s40560-016-0127-6

**Published:** 2016-01-12

**Authors:** Nicole Lefel, Loes Janssen, Jos le Noble, Norbert Foudraine

**Affiliations:** Department of Critical Care, VieCuri Medical Center, Tegelseweg 210, 5912 BL Venlo, The Netherlands; Department of Epidemiology, VieCuri Medical Center, Tegelseweg 210, 5912 BL Venlo, The Netherlands

**Keywords:** Contrast-induced nephropathy, Contrast-induced AKI, Prophylactic therapy, ICU

## Abstract

**Background:**

Intravenously administered iodine-containing contrast medium (CM) is associated with the development of contrast-induced nephropathy (CIN). Data on the effectiveness of sodium bicarbonate therapy in the prevention of CIN are controversial. Furthermore, the incidence of and risk factors for CIN in intensive care unit (ICU) patients are poorly defined. We investigated the effectiveness of sodium bicarbonate prophylaxis and the incidence of and risk factors for CIN in a heterogeneous ICU population.

**Methods:**

This retrospective cohort study included patients admitted to the ICU in 2009–2011 who received CM for computed tomography (CT).

**Results:**

Two hundred eleven CT scans with CM, performed in 170 patients, were included in the study. Contrast prophylaxis with sodium bicarbonate was administered in 48 of the 211 cases. CIN developed in 19 of the 48 cases receiving prophylaxis and in 39 of 163 cases not receiving prophylaxis (*p* = 0.03). In 115 CTs performed in patients with a glomerular filtration rate (GFR) >60 mL/min, prophylaxis was administered 15 times (13 %) and no prophylaxis was administered 100 times (87 %). CIN developed in 12 and 13 % of these cases, respectively (NS). In 96 CTs in patients with a GFR <60 mL/min, 17 of 33 (51.5 %) cases receiving prophylaxis developed CIN and 27 of 63 (42.9 %) cases not receiving prophylaxis developed CIN (NS). Prophylactic sodium bicarbonate therapy did not prevent CIN in our patients, irrespective of pre-existing renal failure. Pre-existing renal impairment (odds ratio 4.41), an elevated Acute Physiology and Chronic Health Evaluation (APACHE) IV score (odds ratio 1.02), and higher haemoglobin levels (odds ratio 0.64) were significant and independent risk factors associated with the development of CIN.

**Conclusions:**

Prophylactic isotonic sodium bicarbonate was not associated with a decreased incidence of CIN in ICU patients. Current sodium bicarbonate prophylaxis guidelines cannot be generalized to a heterogeneous ICU population. Pre-existing renal impairment was associated with the highest CIN risk.

## Background

Depending on the nature and degree of a patient’s comorbidities, the intravenous (IV) administration of iodinated contrast media (CM) is associated with the development of contrast-induced nephropathy (CIN). The incidence of CIN varies between 1 and 50 % depending on patient population, baseline risk factors, and the criteria by which it is defined [1–3]. The definition of CIN includes an exposure to a contrast agent (iodinated CM), an absolute and/or relative increase in serum creatinine (sCr) compared to baseline values following exposure to a contrast agent, a temporal relationship between the rise in sCr and CM exposure, and the exclusion of reasonable alternative explanations for renal impairment [4]. CIN is the third most common cause of hospital-acquired renal failure and accounts for approximately 11 % of renal failure cases [5]. Most research, however, has been conducted in an outpatient cardiology setting, and studies regarding CIN in intensive care unit (ICU) populations are scarce and inconsistent. In previous studies in the ICU population, the incidence of CIN has varied from 14 to 23 %, depending on the definition of CIN used [6–8].

CIN is associated with poor short- and long-term outcomes, including the need for renal replacement therapy (RRT), a longer length of stay in the ICU, and a higher mortality rate [6–9]. A case-matched study by Cely et al. showed that changes in renal function are more likely to be attributable to factors other than the contrast exposure itself [10]. They concluded that persistent (>3 days) renal impairment due to contrast exposure occurred in <2 % of the studied population.

Renal dysfunction in ICU patients occurs according to a multihit model, and most patients have more than one risk factor for renal dysfunction. Reported risk factors for developing CIN in the ICU include diabetes mellitus, sepsis, anaemia, hypotension and hemodynamic failure, administration of potentially nephrotoxic drugs, and renal impairment prior to exposure to a contrast agent [6, 8–10]. The scarcity of data regarding the effectiveness of preventive procedures for CIN in critically ill patients has led to a debate regarding the optimal prophylactic therapy. Therefore, the aim of this study was to assess the effectiveness of the prophylactic administration of an isotonic sodium bicarbonate solution in preventing CIN in a heterogeneous ICU population of patients undergoing a computed tomography (CT) scan with IV CM.

## Methods

### Patient inclusion criteria and data collection

This was a single-site retrospective study in a teaching hospital with a 16-bed general ICU. All ICU patients admitted between January 2009 and December 2011 who received CM for CT imaging were included. Informed consent could not be obtained and was not required by the Ethic Review Board because of the retrospective study design. Nonionic iso-osmolar CM was used in all patients. Patients undergoing RRT at the time of CM administration, patients who underwent a second CT scan within 4 days of the index CT scan, or patients who died within the 4 days following the CT scan were excluded. Serum Cr was recorded daily. All patients were followed for at least 4 days. In case of earlier death, the last available sCr was used.

The primary independent variable was the use of prophylactic treatment. Other variables included the mean sCr from 2 days prior to the index CT and the cumulative fluid balance from 2 days prior to the CT scan. Glomerular filtration rate (GFR) was calculated using age and sCr. Our institution follows a standard protocol for prophylactic treatment of patients at risk of CIN (GFR < 60 mL/min or elevated sCr). The guidelines we followed at the time of the study called for the administration of sodium bicarbonate (8.4 %) 90 mL added to glucose (5 %) 500 mL infused at a rate of 3 mL/kg/h for 1 h prior to CM infusion and at 1 mL/kg/h for 6 h after CM infusion [11].

The primary endpoint was the effectiveness of prophylactic therapy in the prevention of CIN. The secondary endpoints were the incidence of CIN, defined as a rise of ≥44 μmol/L and/or a 25 % increase in baseline sCr within 4 days of CM administration, and the identification of potential risk factors for the development of CIN in ICU patients. To study the effectiveness of prophylactic therapy, results are presented separately for CT scans in patients with GFR >60 mL/min and in patients with GFR <60 mL/min.

### Statistical analysis

Categorical variables were analysed using Pearson’s chi-square test or Fisher’s exact test. A *p* value <0.05 was considered statistically significant. Continuous variables were analysed using an independent *t* test or Mann-Whitney *U* test depending on the distribution of the variable. Multivariate binary logistic regression analysis was performed to assess the risk factors associated with the development of CIN. Apart from prophylactic therapy, all variables that differed significantly between CT procedures in patients who did and did not develop CIN when tested with univariate analysis were included in the multivariate analysis. All analyses were performed using IBM SPSS Statistics, Version 22.0 (IBM Corp, Armonk, NY, USA).

## Results

### Patients

There were 358 CT scans eligible for inclusion. Of these, 140 CT procedures met the a priori exclusion criteria. Additionally, 7 procedures were excluded owing to missing sCr data following the CT scan. Therefore, 211 CT procedures in 170 patients were included in the final analysis. The baseline characteristics of the study population are shown in Table 1. The mean patient age was 65.3 years (range, 27–88 years). Men comprised 59.2 % (125) of the CT procedure patients, and 70.1 % of cases (148) were non-surgical cases. The mean Acute Physiology and Chronic Health Evaluation (APACHE) IV score of all included CT procedures was 79.5 (the predicted in-hospital mortality was 35.6 %).

**Table 1:** Baseline characteristics of the study population

Variable	Total number of CT^*^ scans (*n* = 211)	No prophylaxis (*n* = 163)	Prophylaxis (*n* = 48)	*p* value
Male gender	125 (59.2 %)	99 (60.7 %)	26 (54.2 %)	0.42
Age (years)	65.3 (12.5)	64.3 (12.6)	68.6 (11.5)	0.04
Baseline sCr (μmol/L)	77.7 (71.5)	73.0 (53.0)	104.5 (134.0)	**<** *0.01*
Baseline GFR^$^ (mL/min)	68.6 (37.9)	73.6 (38.4)	51.8 (31.0)	**<** *0.01*
^§^APACHE II score	20.4 (8.3)	20.5 (8.5)	20.0 (7.5)	0.70
APACHE IV score	79.5 (30.1)	78.6 (31.6)	82.5 (24.4)	0.43
Predicted in-hospital mortality (%)	35.6 % (24.2 %)	34.4 (25.0)	39.8 (21.2 %)	0.18
SOFA^#^ day 1	5.0 (6.0)	6.5 (5.0)	5.0 (5.0)	0.07
SOFA day 4	6.0 (5.0)	6.0 (7.0)	4.0 (5.0)	0.02
Comorbidities				
Diabetes	32 (15.2 %)	31 (19.0 %)	1 (2.1 %)	**<** *0.01*
Heart failure	11 (5.2 %)	6 (3.7 %)	5 (10.4 %)	0.09
Sepsis	56 (26.5 %)	42 (25.8 %)	14 (29.2 %)	0.64
Haemoglobin < 6 (mmol/L)	57 (27.0 %)	42 (25.8 %)	15 (31.3 %)	0.45
Fluid balance	2529 (4701)	2401 (4639)	3123 (5695)	0.54
Patients receiving norepinephrine	54 (25.6 %)	43 (26.4 %)	11 (22.9 %)	0.63
Patients receiving aminoglycosides	12 (5.7 %)	10 (6.1 %)	2 (4.2 %)	1.00

Dichotomous data are noted as the number of CT scans with the presence of the concerning variable in that group (percentage of the total number of CTs). Continuous data are noted as the mean (standard deviation) or median (interquartile range)

^*^ *CT* computed tomography, ^$^ *GFR* glomerular filtration rate, *sCr* serum creatinine level, ^§^ *APACHE* Acute Physiology and Chronic Health Evaluation,^#^ *SOFA* Sequential Organ Failure Assessment

### Outcomes

Overall, CIN occurred 58 times (27.5 %) (Table 2). Ninety-six cases undergoing CT procedures had an increased risk of developing acute kidney injury (AKI) before the index CT based on a calculated GFR <60 mL/min. After CM administration, kidney function remained stable in 52 cases (54.2 %), while renal function deteriorated in the other 44 cases (45.8 %). Despite recommendations to administer prophylactic therapy to high-risk patients (including patients with impaired renal function), only 33 (34.4 %) of these 96 CT procedure cases received prophylactic therapy. Of these 33 cases, CIN developed in 17 cases (51.5 %) (Table 2). CIN developed in 27 of the remaining 63 cases (65.6 %) in which no prophylactic therapy was administered (42.9 %, *p* = 0.42). RRT was necessary in 9 cases (9.3 %); 7 (7.3 %) of these cases did not receive prophylactic therapy (*p* = 0.27).

**Table 2:** Effectiveness of sodium bicarbonate prophylaxis for the prevention of contrast-induced nephropathy in cases with or without pre-existing renal impairment

	GFR > 60 mL/min (*n* = 115)			GFR < 60 mL/min (*n* = 96)		
	No prophylaxis (*n* = 100)	Prophylaxis (*n* = 15)	*p* value	No prophylaxis (*n* = 63)	Prophylaxis (*n* = 33)	*p* value
No CIN	88 (88.0 %)	13 (86.7 %)	1.00	36 (57.1 %)	16 (48.5 %)	0.42
CIN	12 (12.0 %)	2 (13.3 %)		27 (42.9 %)	17 (51.5 %)	
RRT/CIN	3/12 (25.0 %)	0/2 (0.0 %)	1.00	7/27 (25.9 %)	2/17 (11.8 %)	0.27

Dichotomous data are noted as the number of CT scans with the presence of the concerning variable in that group (percentage of the total number of CT scans)

*CIN* contrast-induced nephropathy, *RRT* renal replacement therapy, *GFR* glomerular filtration rate

One hundred fifteen cases had normal renal function prior to CM administration based on a calculated GFR >60 mL/min, and therefore, their risk of developing AKI was surmised to be low. Fourteen (12.2 %) of these 115 cases developed CIN. Fifteen cases (13.0 %) with normal renal function received prophylactic therapy; 2 (13.3 %) of these cases developed CIN nevertheless (Table 2). Of the 100 cases in which no prophylactic therapy was administered, CIN developed 12 times (12.0 %) and RRT was necessary in 3 (25.0 %) of these cases. The occurrence of CIN was not statistically different between patients with normal renal function undergoing CT scans with or without prophylactic therapy (*p* = 1.00).

A comparison of the characteristics of those cases with CIN vs. those without the development of CIN is displayed in Table 3. Overall, 39 patients died. The mortality rate was significantly higher in patients who developed CIN than in those who did not (37.0 vs. 6.3 %, *p* < 0.01). Causes of death are shown in Table 4.

**Table 3:** Characteristics of patients who did or did not develop contrast-induced nephropathy

	No CIN	CIN	*p* value
Number of CT scans	153 (72.5 %)	58 (27.5 %)	
Age (years)	64 (13)	69 (10)	<0.01
Male gender	88 (57.5 %)	37 (63.8 %)	0.41
Diabetes mellitus	22 (14.4 %)	10 (17.2 %)	0.61
Heart failure	6 (3.9 %)	5 (8.6 %)	0.18
Sepsis	41 (26.8 %)	15 (25.9 %)	0.89
Baseline GFR < 60 mL/min	52 (34.0 %)	44 (75.9 %)	<0.01
Haemoglobin (mmol/L)	6.6 (1.1)	6.2 (0.88)	0.01
APACHE II score	19.5 (7.9)	22.8 (8.9)	<0.01
APACHE IV score	74.5 (29.1)	92.6 (28.9)	<0.01
SOFA day 1	8.0 (7.0)	5.0 (4.0)	<0.01
SOFA day 4	11.0 (6.0)	4.0 (3.0)	<0.01
Fluid balance (mL/24 h)	2180 (4566)	3488 (7251)	0.03
Aminoglycoside (single-dose) use	5 (3.3 %)	7 (12.1 %)	0.02
Norepinephrine use	32 (20.9 %)	22 (37.9 %)	0.01
Prophylactic therapy	29 (19.0 %)	19 (32.8 %)	0.03

Dichotomous data are noted as the number of patients with the presence of the concerning variable in that group (percentage of the entire population). Continuous data are noted as the mean (standard deviation) or median (interquartile range)

*CIN* contrast-induced nephropathy, *CT* computed tomography, *GFR* glomerular filtration rate, *APACHE* Acute Physiology and Chronic Health Evaluation, *SOFA* Sequential Organ Failure Assessment

**Table 4:** Causes of mortality

Cause	Prophylaxis		Total
	Yes	No	
MODS	12	6	18
Sepsis	8	4	12
Respiratory insufficiency	3	0	3
Anoxic encephalopathy	2	0	2
Bleeding	2	0	2
Combined respiratory and cardiac failure	0	2	2
Total	27	12	39

*MODS* multiple organ dysfunction syndrome

No significant differences were found between patients with or without prophylaxis

A multivariate logistic regression analysis identified pre-existing renal impairment (GFR < 60 mL/min) (odds ratio 4.58, *p* < 0.01), a higher APACHE IV score (odds ratio 1.02, *p* = 0.02), and a reduced haemoglobin level below 6 mmol/L (odds ratio 0.64, *p* = 0.03) as independent risk factors for CIN (Table 5). Single-dose aminoglycoside administration revealed a strong trend towards an independent risk factor in our study (odds ratio 3.87, *p* = 0.05), but the results did not reach a level of statistical significance. A positive fluid balance, no norepinephrine use, and increased age were associated with the CIN group with univariate analysis (*p* = 0.03, *p* = 0.01, and *p* < 0.01, respectively). However, in the multivariate analysis, these variables were not statistically significant independent risk factors for the development of CIN.

**Table 5:** Risk factors for contrast-induced nephropathy in intensive care unit patients (multivariate logistic regression analysis)

Variable	OR	*p* value
Prophylactic pre- and post-hydration (Y/N)	1.32	0.49
GFR < 60 mL/min or >60 mL/min	4.41	<0.01
APACHE IV score (per increasing point)	1.02	0.02
Norepinephrine use (Y/N)	1.49	0.32
Aminoglycoside use (Y/N)	3.87	0.05
Fluid balance (per ml)	1.00	0.95
Haemoglobin (>mmol/L)	0.64	0.03
Age (/year)	1.02	0.37

All variables that differed significantly between the no CIN and the CIN groups in a univariate test (Table 4) were included in the multivariate regression analysis, except for the APACHE II score. This score is highly correlated with APACHE IV and therefore was discarded from the multivariate analysis

*GFR* glomerular filtration rate, *OR* odds ratio, *APACHE* Acute Physiology and Chronic Health Evaluation

## Discussion

The main finding of this study was the lack of an association between the administration of prophylactic isotonic sodium bicarbonate infusion and the prevention of CIN in ICU patients receiving a contrast agent for CT imaging. These findings are similar to recent studies by Au et al. [12] and Deek et al. [13].

A secondary endpoint of our study was the evaluation of potential risk factors for the development of CIN in ICU patients. Our findings indicate that pre-existing renal impairment (GFR < 60 mL/min), a high APACHE IV score, and haemoglobin are significant independent risk factors for the development of CIN (*p* < 0.01, *p* = 0.02, *p* = 0.03, respectively). GFR was chosen for the assessment of renal impairment in our study because baseline sCr measurements were not always available and because urinary output can decline due to other factors, such as hypotension or hypovolemia. Additionally, generally accepted protocols recommend the use of prophylactic therapy based on a GFR <60 mL/min.

Remarkably, patients with no apparent renal dysfunction (GFR > 60 mL/min) are also at risk for CIN. It is not known if prophylactic therapy can prevent CIN in these patients. The number of CT procedures in our study was too low to draw unequivocal conclusions, but our findings indicate no correlation between pre-diagnostic bicarbonate administration and CIN incidence.

Prophylactic bicarbonate therapy addresses radiographic CM administration, which is only one possible risk factor for the development of renal dysfunction. ICU patients frequently develop renal dysfunction, even in the absence of CM administration, and not all patients with pre-existing renal dysfunction experienced further deterioration of their renal function after contrast administration. A multihit model may explain the lack of efficacy of prophylactic therapy in the prevention of CIN. Heart failure, anaemia, sepsis, hypoxia, and medications can all negatively affect kidney function. In our ICU patients, we found haemoglobin and APACHE scores independently associated with CIN, and an additional insult, such as the use of CM, may act synergistically with these other factors to cause more overt AKI.

We believe that generally accepted protocols do not apply to ICU patients who are exposed to multiple potential risk factors for renal impairment. Another reason for the lack of efficacy of prophylaxis in ICU patients may be related to the timing or dosage of the contrast agent. However, the amount of contrast used in our study (80 mL) is comparable to the amount typically used in coronary angiography patients. Although there is no evidence to support the inferiority of sodium bicarbonate 8.4 % to sodium chloride 0.9 % as prophylactic therapy in the prevention of CIN, the dose of bicarbonate used for prophylaxis may potentially be too low to counterbalance a contrast-induced kidney injury [14]. Moreover, the overall effectiveness of any prophylactic effect of sodium bicarbonate or sodium chloride is still under debate and the object of ongoing study [15–18].

The prevalence of diabetes was slightly different between patients who received prophylactic therapy and those who did not, and this confounding factor may have influenced our findings. No other baseline factors were significantly different between those receiving prophylactic therapy and those who did not receive prophylactic therapy. However, diabetes is not a known independent risk factor for the development of CIN and was not identified as a potential risk factor in our multivariate analysis. Nevertheless, other hidden confounders may have influenced our results.

Aminoglycosides are strongly associated with renal dysfunction, especially in patients with persistently high through levels. In our study, the administration of even a single dose of an aminoglycoside (3 mg/kg) appeared to increase the risk of developing CIN; however, this risk was of borderline statistical significance (*p* = 0.05).

Another secondary endpoint was to determine the incidence of CIN. The incidence of CIN in our study was 27.5 %. We chose to use a relatively broad definition of CIN. Instead of measuring sCr within 48 h, which is commonly used, we included measurements up to 96 h after the administration of the contrast agent to ensure that cases of late-onset CIN were not missed. The Kidney Disease/Improving Global Outcomes (KDIGO) guidelines, which were used to establish a definition for contrast-induced acute kidney injury (CI-AKI), acknowledge the fact that a consistent time course for the diagnosis of CIN has yet to be determined. Studies have shown a rise in sCr up to 5 days after contrast administration [19]. The higher incidence of CIN found in our study may be due to the different definitions of CIN used and/or to variations in the patient population studied [20].

Limitations of our study include the sample’s size and retrospective design. Owing to the relatively small number of subjects, a reliable subgroup statistical analysis was not possible. Regarding the study design, a prospective randomized controlled trial is urgently needed but would be challenging to perform because of comorbidities and timing implications including the time of diagnosis, time of CT scanning, and timing of prophylactic therapy.

## Conclusions

In our study, prophylactic therapy with sodium bicarbonate was not associated with the prevention of CIN. Pre-existing renal impairment (GFR < 60 mL/min), a high APACHE IV score at the time of admission, and anaemia were significant risk factors for CIN. Aminoglycoside use was a borderline statistically significant risk factor (*p* = 0.05), but more research is needed. The incidence of CIN in this study was 27.5 %, indicating that CIN occurs frequently in ICU patients, even when renal function appears normal. Continued studies to improve methods of prophylaxis are required to improve the short- and long-term outcomes for ICU patients.

## Abbreviations

AKI: acute kidney injury
APACHE: Acute Physiology and Chronic Health Evaluation
CI-AKI: contrast-induced acute kidney injury
CIN: contrast-induced nephropathy
CM: contrast media
GFR: glomerular filtration rate
ICU: intensive care unit
KDIGO: Kidney Disease/Improving Global Outcomes
MODS: multiple organ dysfunction syndrome
RRT: renal replacement therapy
sCr: serum creatinine
SOFA: Sequential Organ Failure Assessment
